# Post-radioembolization yttrium-90 PET/CT - part 1: diagnostic reporting

**DOI:** 10.1186/2191-219X-3-56

**Published:** 2013-07-25

**Authors:** Yung-Hsiang Kao, Jeffrey D Steinberg, Young-Soon Tay, Gabriel KY Lim, Jianhua Yan, David W Townsend, Angela Takano, Mark C Burgmans, Farah G Irani, Terence KB Teo, Tow-Non Yeow, Apoorva Gogna, Richard HG Lo, Kiang-Hiong Tay, Bien-Soo Tan, Pierce KH Chow, Somanesan Satchithanantham, Andrew EH Tan, David CE Ng, Anthony SW Goh

**Affiliations:** 1 Department of Nuclear Medicine and PET, Singapore General Hospital, Outram Road, Singapore 169608, Singapore; 2 Department of Nuclear Medicine, Sir Charles Gairdner Hospital, Hospital Ave, Perth, Western Australia 6009, Australia; 3 Department of Nuclear Medicine, Austin Hospital, 145 Studley Rd, Melbourne, VIC 3084, Australia; 4 Singapore Bioimaging Consortium, Agency for Science Technology and Research (A*STAR), 11 Biopolis Way, Helios Building, Singapore 138667, Singapore; 5 Agency for Science Technology and Research (A*STAR) - National University of Singapore (NUS) Clinical Imaging Research Centre, 14 Medical Drive, Singapore 117599, Singapore; 6 Department of Pathology, Singapore General Hospital, Outram Road, Singapore 169608, Singapore; 7 Department of Diagnostic Radiology, Singapore General Hospital, Outram Road, Singapore 169608, Singapore; 8 Department of General Surgery, Singapore General Hospital, Outram Road, Singapore 169608, Singapore; 9 Department of Surgical Oncology, National Cancer Centre Singapore, 11 Hospital Drive, Singapore 169610, Singapore; 10 Office of Clinical Sciences, Duke-National University of Singapore Graduate Medical School, 8 College Rd, Singapore 169857, Singapore; 11 Department of Radiology, Leiden University Medical Center, Albinusdreef 2, 2300 RC, Leiden, The Netherlands

**Keywords:** Yttrium-90 radioembolization, Selective internal radiation therapy, Yttrium-90 PET/CT, Bremsstrahlung SPECT/CT, Diagnostic reporting, Non-target activity

## Abstract

**Background:**

Yttrium-90 (^90^Y) positron emission tomography with integrated computed tomography (PET/CT) represents a technological leap from ^90^Y bremsstrahlung single-photon emission computed tomography with integrated computed tomography (SPECT/CT) by coincidence imaging of low abundance internal pair production. Encouraged by favorable early experiences, we implemented post-radioembolization ^90^Y PET/CT as an adjunct to ^90^Y bremsstrahlung SPECT/CT in diagnostic reporting.

**Methods:**

This is a retrospective review of all paired ^90^Y PET/CT and ^90^Y bremsstrahlung SPECT/CT scans over a 1-year period. We compared image resolution, ability to confirm technical success, detection of non-target activity, and providing conclusive information about ^90^Y activity within targeted tumor vascular thrombosis. ^90^Y resin microspheres were used. ^90^Y PET/CT was performed on a conventional time-of-flight lutetium-yttrium-oxyorthosilicate scanner with minor modifications to acquisition and reconstruction parameters. Specific findings on ^90^Y PET/CT were corroborated by ^90^Y bremsstrahlung SPECT/CT, ^99m^Tc macroaggregated albumin SPECT/CT, follow-up diagnostic imaging or review of clinical records.

**Results:**

Diagnostic reporting recommendations were developed from our collective experience across 44 paired scans. Emphasis on the continuity of care improved overall diagnostic accuracy and reporting confidence of the operator. With proper technique, the presence of background noise did not pose a problem for diagnostic reporting. A counter-intuitive but effective technique of detecting non-target activity is proposed, based on the pattern of activity and its relation to underlying anatomy, instead of its visual intensity. In a sub-analysis of 23 patients with a median follow-up of 5.4 months, ^90^Y PET/CT consistently outperformed ^90^Y bremsstrahlung SPECT/CT in all aspects of qualitative analysis, including assessment for non-target activity and tumor vascular thrombosis. Parts of viscera closely adjacent to the liver remain challenging for non-target activity detection, compounded by a tendency for mis-registration.

**Conclusions:**

Adherence to proper diagnostic reporting technique and emphasis on continuity of care are vital to the clinical utility of post-radioembolization ^90^Y PET/CT. ^90^Y PET/CT is superior to ^90^Y bremsstrahlung SPECT/CT for the assessment of target and non-target activity.

## Background

The science of yttrium-90 (^90^Y) radioembolization is rapidly evolving. In suitable patients, ^90^Y radioembolization is an effective treatment modality for inoperable liver malignancies, and its expansion into organs other than the liver has been described [1-5]. Contemporary techniques and clinical outcomes are well described in recent literature [6-11]. ^90^Y radioembolization is complex and requires close multi-disciplinary coordination across all stages of its workflow, described as a *continuum* of care [7].

^90^Y radioembolization is brachytherapy delivered by ^90^Y-labelled microspheres implanted permanently in the target vascular bed following intra-arterial injection. The microsphere biodistribution within the target arterial territory is dependent on the locoregional flow environment distal to the point of injection. This in turn is influenced by a myriad of inter-related biophysical variables such as the catheter tip cross-sectional spatial location, injection rate and timing interval, proximity to branching daughter vessels, extent of shunting, cardiovascular status, particle load and microembolization [12-14]. Such complexity means that technical success should not be assumed without some form of microsphere imaging. A technically unsuccessful ^90^Y radioembolization may have prognostic implications for tumor response or non-target radiation injury.

In recent years, ^90^Y bremsstrahlung single-photon emission computed tomography with integrated computed tomography (SPECT/CT) has been the modality-of-choice for post-radioembolization microsphere imaging, for the lack of a better alternative [8]. ^90^Y bremsstrahlung scintigraphy suffers from a low spatial resolution, ranging between 11 to 15 mm depending on the choice of energy window, collimator, and image processing [15-17]. This is due to its inherent technical limitation as a form of indirect imaging of a continuous scatter radiation spectrum without a pronounced photopeak, resulting in a coarse representation of the microsphere biodistribution. ^90^Y bremsstrahlung activity on SPECT/CT often appears smooth and diffuse, with ill-defined margins which are difficult to distinguish from closely adjacent activity foci. As a result, assessment of activity within subcentimeter tumors or tumor vascular thrombosis are often suboptimal on ^90^Y bremsstrahlung SPECT/CT. Quantitatively, ^90^Y bremsstrahlung scintigraphy is largely inaccurate despite compensation techniques for attenuation, scatter, and collimator-detector response, making it unsuitable for dose–response analysis [18-20].

Positron emission tomography with integrated computed tomography (PET/CT) represents a technological leap from ^90^Y bremsstrahlung SPECT/CT, both qualitatively and quantitatively, allowing direct imaging of ^90^Y microspheres. Although ^90^Y is predominantly a β^−^ emitter, coincidence imaging of ^90^Y is possible because of a minor decay branch to the 0^+^ first excited state of zirconium-90 followed by β^−^β^+^ internal pair production at a very low branching ratio of 31.86 ± 0.47 × 10^−6^[21-24]. However, the clinical application of ^90^Y PET/CT has been fraught with challenges. Today’s time-of-flight PET/CT scanners use lutetium-based crystals due to its superior timing resolution. Such crystals, such as lutetium-yttrium-oxyorthosilicate (LYSO), contain 2.6% of naturally occurring lutetium-176 (^176^Lu) which results in a background activity of intrinsic true and random coincidences which cannot be distinguished from coincidences by an external radioactive source [22,25]. This issue was previously thought to be clinically irrelevant until recent interest in ^90^Y PET/CT. In addition, the low positron activity of ^90^Y and the intrinsic activity of ^176^Lu can have an impact on scatter correction. For time-of-flight PET, several authors have computed the scatter based on the PET activity [26-28]. Since the scatter correction algorithm depends on the PET activity map, which is poor due to low signal and intrinsic noise, scatter correction will be negatively affected. The unfavorable combination of intrinsic background activity and a very low positron fraction results in reconstructed ^90^Y PET/CT images which at the outset, seem uninterpretable due to high background noise.

Despite this, several institutions have published encouraging early experiences using ^90^Y time-of-flight PET for post-radioembolization imaging [29-31]. These early reports suggest that despite background noise, it is possible to obtain high-resolution images of microsphere biodistribution using conventional time-of-flight PET scanners, with only minor adjustments to scan technique and image reconstruction such as PET acquisition time, number of bed positions, reconstruction iterations, and subsets. ^90^Y phantom studies of lutetium-based time-of-flight PET/CT systems found the spatial resolution to be 9.3 mm, and the minimum detectable ^90^Y radioconcentration of a 10-mm phantom sphere was approximately 1 MBq/ml [23,32]. Count rate linearity was maintained when tested up to 4.7 GBq, and detector saturation from ^90^Y bremsstrahlung radiation was not an issue [22,23,32-34].

For tumor dose quantification, several phantom studies using various protocols on lutetium-based time-of-flight PET/CT systems have recently been published. Carlier et al. found activity quantification of phantom spheres to be feasible at ^90^Y radioconcentrations greater than 2 MBq/ml, with recovery coefficients of approximately 0.6 and 0.7 for spheres of diameters 17 and 28 mm respectively [32]. Willowson et al. showed a phantom sphere of ^90^Y radioconcentration 3.9 MBq/ml and diameter 37 mm to have a recovery coefficient of approximately 0.8, and a trend was observed for recovery coefficients to approach 1 with increasing sphere size [33]. Goedicke et al. achieved a mean quantitative error of +2.4% ± 3.4% standard deviation in a 350 ml phantom, and ^90^Y radioconcentrations greater than 2.9 MBq/ml had quantitative errors less than 2% [34]. Elschot et al. found the absorbed dose quantitative error of a 37-mm diameter phantom sphere of ^90^Y radioconcentration 2.4 MBq/ml to be −11% uncorrected and +6% corrected for partial volume effects [35]. These phantom studies suggest that tumor dose quantification by ^90^Y PET may be feasible, and complements clinical reports of ^90^Y PET tumor dose quantification correlating well with treatment response [24,36,37].

On the basis of superior image resolution, quantitative capability, and encouraged by favorable early experiences [29-31], we recently introduced post-radioembolization ^90^Y PET/CT as an adjunct to ^90^Y bremsstrahlung SPECT/CT in diagnostic reporting. This is a two-part retrospective report reviewing our experience over a 1-year period: Part 1 focuses on qualitative analysis and diagnostic reporting; Part 2 analyzes tissue dose–response by ^90^Y PET quantification and evaluates the accuracy of tumor predictive dosimetry [38].

## Methods

Institutional review board approval was obtained for the conduct and publication of this retrospective report (CIRB 2010/781/C, SingHealth, Singapore), and all research is in compliance with the Helsinki Declaration. We recently introduced ^90^Y PET/CT as an adjunct to ^90^Y bremsstrahlung SPECT/CT for high resolution imaging of microsphere biodistribution. Pairing ^90^Y PET/CT with ^90^Y bremsstrahlung SPECT/CT enabled the trouble shooting of indeterminate findings on either modality, and facilitated the learning of our nuclear medicine physicians in the diagnostic reporting of ^90^Y PET/CT. As there were no ^90^Y PET/CT protocols specific to our scanner make and model in prior literature, our early scan protocols were based on publications of other scanner types [22,29]. In the first few months after implementation, our ^90^Y PET/CT protocol was continually refined by empirical adjustment of acquisition and reconstruction parameters, with a primary emphasis on visual image quality. The aim was to qualitatively achieve a balance between image resolution and background noise, of sufficient clarity for diagnostic reporting purposes. Quantitative accuracy was not a consideration during this early optimization phase.

Our PET/CT is the GE Discovery 690 (General Electric Medical Systems, GEMS, Milwaukee, WI, USA). It uses cerium-activated LYSO crystals, and its image reconstruction is a fully three-dimensional (3D) ordered subset expectation maximization (3D-OSEM) algorithm which incorporates time-of-flight and point spread function information (GEMS name: VUE-point FX; Sharp-IR) [25]. The resulting image is corrected for attenuation, scatter, randoms, dead time, and normalization [25]. ^90^Y PET was acquired using positron fraction 3.186 × 10^−5^, half-life 64.1 h, 15 min per bed position, 1 to 2 bed positions from the diaphragm downwards to cover the entire liver, reconstructed with 3 iterations and 18 subsets. Our full protocol is presented in Additional file 1. ^90^Y bremsstrahlung SPECT/CT was performed on a Philips Precedence (Philips, Amsterdam, The Netherlands), which has a dual-head gamma camera and 16-slice CT. A medium energy general purpose collimator was used. Based on our phantom studies, we centered our energy window at 80% ± 15% keV to capture the most prominent photopeak amidst a continuous bremsstrahlung spectrum. Scans were acquired in 128 frames over 360°, 20 s per frame; 3D-OSEM reconstruction using Philips Astonish software; 3 iterations and 8 subsets; matrix size 128 × 128; low-dose CT for anatomical correlation and attenuation correction. All paired ^90^Y PET/CT and ^90^Y bremsstrahlung SPECT/CT scans were performed within 1 h of each other.

Diagnostic reporting for all paired scans were done in consensus by an experienced team of nuclear medicine physicians with cumulative experience of more than 200 ^90^Y radioembolization cases over 5 years. As no prior guidelines existed in literature for the diagnostic reporting of post-radioembolization ^90^Y PET/CT, a gestalt interpretation was applied to all paired scans based on a thorough knowledge of the case-specific treatment intent, angiographic and dosimetric factors. ^90^Y resin microspheres (SIR-Spheres, Sirtex Medical Limited, New South Wales, Australia) were used. Our predictive dosimetric protocol was previously described, which involves catheter-directed CT angiography for arterial territory delineation, quantitative ^99m^Tc macroaggregated albumin (MAA) SPECT/CT of the target organ, and Medical Internal Radiation Dose (MIRD) macrodosimetry to achieve artery-specific personalized radiation planning [11]. As our treatment paradigm deviates considerably from conventional semi-empirical methods, our nuclear medicine team emphasizes continuity of care and an in-depth knowledge of case-specific angiography, in close collaboration with our interventional radiologists.

One of the qualitative study objectives was to compare the ability of ^90^Y PET/CT versus ^90^Y bremsstrahlung SPECT/CT in providing conclusive information about the presence or absence of ^90^Y activity within targeted tumor vascular thrombosis. We chose to evaluate tumor vascular thrombosis because these are tumorous lesions within small-to-medium calibre vessels which carry prognostic significance [9]. Another qualitative study objective was to compare the ability of ^90^Y PET/CT versus ^90^Y bremsstrahlung SPECT/CT in detecting non-target activity.

Where these were appropriate, specific findings on ^90^Y PET/CT were corroborated by ^90^Y bremsstrahlung SPECT/CT, ^99m^Tc MAA SPECT/CT, follow-up diagnostic sectional imaging (e.g. CT or MRI), or review of clinical records for symptoms or endoscopy. Clinical toxicities attributable to ^90^Y radioembolization were graded according to the Common Terminology Criteria for Adverse Events version 4.03 (CTCAE; National Institutes of Health, National Cancer Institute, USA). A retrospective review of clinical records was performed for all patients to exclude any clinically significant non-target activity undetected by both imaging modalities. The accuracies of both modalities were calculated by binary classification testing.

Our methods for the diagnostic reporting of target and non-target activity are presented in Table 1. In the absence of established diagnostic reporting guidelines, our methods were heuristically formulated based on rational angiographic and dosimetric principles from our collective experience across all paired scans. Using these guidelines, we were able to overcome the problem of background noise and other general issues related to the complexity of ^90^Y radioembolization. Thus, the presence of background noise did not pose a problem for diagnostic reporting. In Table 1, we propose an unconventional technique for non-target activity detection, which counter-intuitively requires an *increase* in noise levels by adjusting the visual display threshold, but was effective in overcoming the problem of background noise.

**Table 1:** **Recommendations for diagnostic reporting of post-radioembolization**^**90 **^**Y PET/CT**

**Item**	**Recommendation**
Continuity of care	Post-radioembolization ^90^Y PET/CT is best reported by the same attending nuclear medicine physician who has followed through the entire planning-therapy continuum from exploratory angiography, predictive dosimetry, to ^90^Y radioembolization.
PET display threshold setting for non-target ^90^Y activity detection	For detection of non-target ^90^Y activity, the operator should actively adjust the upper PET visual display threshold setting to deliberately *increase* the background noise to moderate levels.
Criteria for technical success	1. ^90^Y activity present in the majority of targeted tumors, or good overall activity coverage of large targeted tumors; *and*
	2. The absence of clinically significant non-target ^90^Y activity; *and*
	3. All findings are in keeping with pre-therapy radiation planning expectations.
Criteria for a technically unsuccessful ^90^Y radioembolization	1. The complete, or near-complete absence of ^90^Y activity in the majority of targeted tumors, or poor overall activity coverage of large targeted tumors; *or*
	2. The presence of any non-target ^90^Y activity where ^90^Y PET dose quantification predicts a high likelihood of clinically significant radiation toxicity; *or*
	3. Any other situation where the ^90^Y activity biodistribution is adversely inconsistent with pre-therapy radiation planning expectations.
Criteria for non-target ^90^Y activity	1. Non-random pattern of activity distribution; *and*
	2. Conforms morphologically to an untargeted anatomical structure on CT; *with or without*
	3. A plausible vascular etiology to account for its presence.
Criteria for noise spikes	1. Small, discrete, ovoid activity foci; *and*
	2. Random pattern of distribution which do not conform to underlying anatomy on CT; *and*
	3. No plausible vascular etiology to account for its presence.

As outlined in Table 1, operator adjustment of the PET visual display threshold was a key step to qualitatively distinguish between signal and noise for diagnostic reporting. The lower PET visual display threshold was set to zero for both target and non-target activity detection. For target activity detection, the upper PET visual display threshold setting was adjusted to reduce the background noise to a minimum. On our system, we used the following upper PET visual display threshold settings for target activity detection: median 53% (7,000 kBq/ml), mean 54% (6,900 kBq/ml), 95% confidence interval (CI) 47% to 61% (5,900 to 7,900 kBq/ml).

For non-target activity detection, the upper PET visual display threshold setting was adjusted to deliberately *increase* the background noise to moderate levels. This counter-intuitive technique was necessary because genuine trace non-target activity may appear visually less intense than noise spikes (see “Discussion”). Therefore, if the upper PET visual display threshold had remained at the settings used to suppress background noise for target activity assessment, it will be unlikely for visually subtle, trace non-target activity to be detected by the operator. Albeit subjective, this method improved the detection of non-target activity with appropriate diagnostic technique (Table 1). On our system, we used the following upper PET visual display threshold settings for non-target activity detection: median 14% (2,000 kBq/ml), mean 22% (2,200 kBq/ml), 95% CI 4 to 41% (1,200 to 3,100 kBq/ml). Non-target activity was sometimes more easily detected on the rotating maximum intensity projection (MIP) than on the sectional PET or PET/CT images. On the rotating MIP, any activity in a non-random pattern protruding from the smooth outline of targeted tissue amidst background noise was regarded as suspicious and pursued on sectional PET and PET/CT images. The above data is tabulated in Additional file 1: Table S1.

### Terms and definitions

The following terms and definitions are used throughout our two-part report. *Predictive dosimetry* refers to personalized radiation planning guided by modern tomographical techniques of microsphere simulation, where intended absorbed doses are determined a priori based on case-specific clinical, angiographic and dosimetric parameters, in accordance with the achievable intent [11]. *Target* refers to the arterial territory intended for ^90^Y radioembolization, represented by planning target volumes used in artery-specific predictive dosimetry [11]. *Non*-*target* refers to unintended ^90^Y activity beyond planning target volumes, e.g., stomach and duodenum, which also includes untargeted non-tumorous liver due to microsphere reflux or arterio-portal shunting. *Technical success* refers to the qualitative assessment of a satisfactory ^90^Y activity biodistribution in accordance with radiation planning expectations as seen on post-radioembolization ^90^Y PET/CT, not to be confused with ‘clinical success’ [8]. Our definition of technical success is slightly dissimilar to conventional reporting standards [8] because angiographic appearances alone at the conclusion of ^90^Y radioembolization may not necessarily reflect the actual microsphere biodistribution [12,14]. The *planning*-*therapy continuum* for ^90^Y radioembolization at our institution is a seamless, integrated workflow for nuclear medicine physicians with emphasis on continuity of care, a multi-disciplinary approach, open and continuous inter-disciplinary communication, and predictive dosimetry.

## Results and discussion

### Results

A total of 63 ^90^Y radioembolization procedures were performed over a 1-year period. Of these, 44 (70%) paired ^90^Y PET/CT and ^90^Y bremsstrahlung SPECT/CT scans were performed. Twenty-one of 44 paired scans were performed using various ^90^Y PET/CT protocols during our early optimization phase. After finalization of our scan protocol, a further 23 paired scans were performed. For data consistency, all case examples, statistical and dose–response results presented in this two-part report were limited to the latter 23 patients only.

Of these 23 patients, 19 (83%) had hepatocellular carcinomas (HCC), 2 had intra-hepatic cholangiocarcinomas, 1 had liver metastases from pancreatic neuroendocrine cancer, and 1 had a bulky right adrenal gland metastasis from gastrointestinal stromal tumor (GIST) - a case of ^90^Y radioembolization of an organ other than the liver. Median age was 64.5 years (mean 63.5 years; range 40 to 77 years); there were 16 (70%) male patients. Tumor vascular thrombosis was present in seven (30%) patients: portal vein in six patients and inferior vena cava in one; and all were HCCs. The median hepatopulmonary shunt fraction estimated by ^99m^Tc MAA planar scintigraphy was 5.8% (mean 6.6%; range 1.5% to 24.2%).

A total of 39 arterial territories were treated across 23 patients (mean 2; range 1 to 4), which included the right inferior phrenic, right internal mammary, and right inferior adrenal arteries. The median injected ^90^Y activity was 2.2 GBq (mean 2.4 GBq; range 1.1 to 5.0 GBq), and the median time interval from ^90^Y radioembolization to ^90^Y PET/CT was 21.3 h (mean 19.2 h; range 4.7 to 24.0 h). One patient was deemed technically unsuccessful due to non-target activity in the gastric and duodenal walls resulting in CTCAE Grade 3 toxicity; all others were technically successful. All the above data are summarized in Table 2 and presented in full detail in Additional file 1: Tables S2 and S3.

**Table 2:** Patient and treatment characteristics

**Characteristic**	**Data**
Sex (*n*)	
Male	16
Female	7
Age (years)	
Median	64.5
Range	40 to 77
Disease (*n*)	
Hepatocellular carcinoma	19
Cholangiocarcinoma	2
Pancreatic neuroendocrine	1
Adrenal metastatic GIST	1
Tumor vascular thrombosis (*n*)	
Hepatic portal vein	6
Inferior vena cava	1
Treatment extent (*n*)	
Liver	22
Whole-liver	9
Lobar	11
Segmental	2
Organ other than liver	1
No. of arterial territories (*n*)	
1	11
2	9
3	2
4	1
Lung shunt (%)	
Median	5.8
Range	1.5 to 24.2
Total injected ^90^Y activity (GBq)	
Median	2.2
Range	1.1 to 5.0
Interval from radioembolization to PET (h)	
Median	21.3
Range	4.7 to 24.0

**Figure 1:**
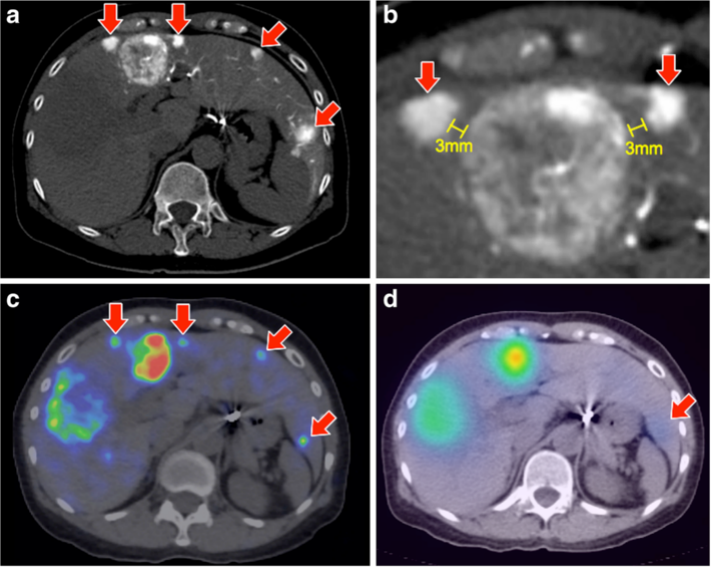
**Patient 23. (a)** Multifocal HCC seen on catheter-directed CT angiogram of the replaced left hepatic artery, arising from the left gastric artery. Four small tumors are indicated by arrows. **(b)** Enlarged view of segment IV shows the larger central tumor being flanked by two smaller, closely adjacent tumors separated by approximately 3 mm. **(c)** Post-radioembolization ^90^Y PET/CT confirms focal activity within the four small tumors, depicted in high resolution. **(d)**^90^Y bremsstrahlung SPECT/CT was barely able to detect subtle activity in the larger (arrow) of the four small tumors, but was unable to confirm any focal activity within the other three small tumors due to its low resolution.

**Figure 2:**
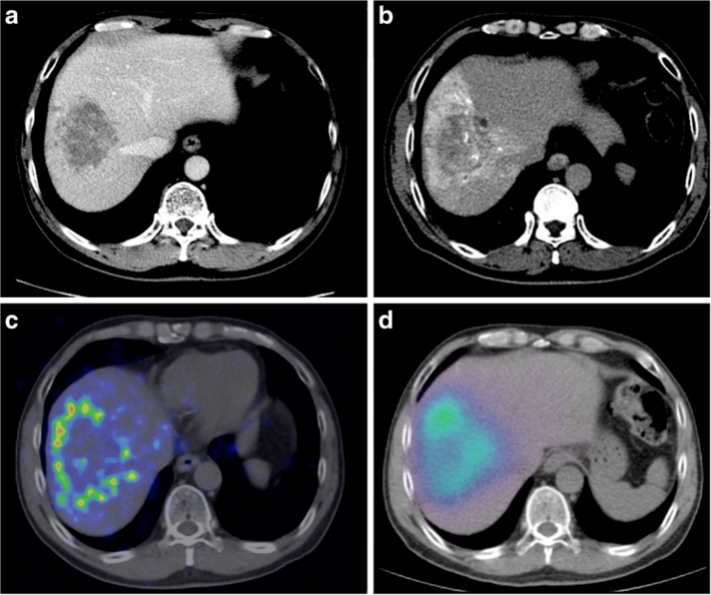
**Patient 20. (a)** Large hypovascular HCC in the right lobe, seen on contrast-enhanced CT Abdomen. **(b)** Catheter-directed CT angiogram of the right hepatic artery demonstrates tumor hypovascularity and delineates the target arterial territory. **(c)**^90^Y PET/CT depicts, in high resolution, a rim-like activity in the tumor periphery with a large area of relative central photopenia, typical of hypovascular tumors. **(d)**^90^Y bremsstrahlung SPECT/CT poorly demonstrates the peripheral activity due to its low image resolution and under-represents the extent of central photopenia.

**Figure 3:**
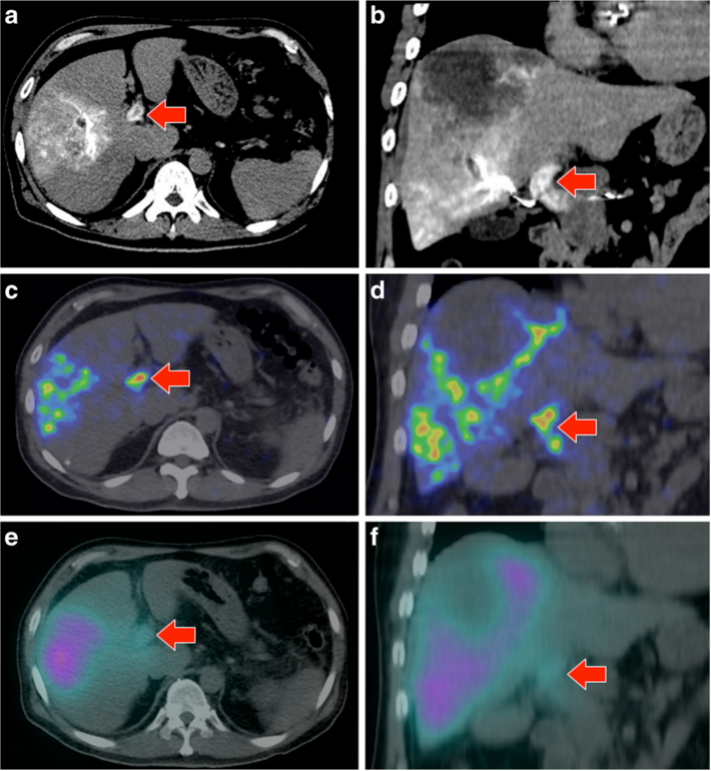
**Patient 10.** Portal vein tumor thrombosis in a large heterogeneous HCC. **(a**, **b)** Catheter-directed CT angiogram of the right hepatic artery demonstrates contrast enhancement within portal vein tumor thrombosis (arrow). **(c**, **d)**^90^Y PET/CT depicts focal ^90^Y activity within the portal vein tumor thrombus in high resolution. **(e**, **f)** The same activity is poorly visualized on ^90^Y bremsstrahlung SPECT/CT as subtle, ill-defined activity.

**Figure 4:**
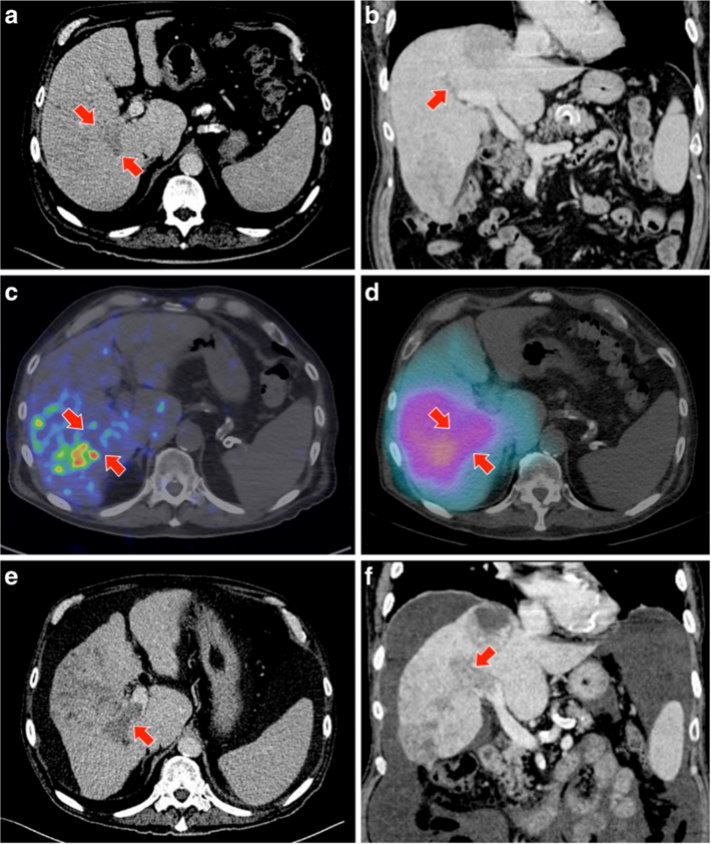
**Patient 12. (a**, **b)** Portal vein tumor thrombosis (arrow) in a multifocal HCC seen on portal venous phase of a triphasic CT liver. **(c)**^90^Y PET/CT demonstrates absent activity within the portal vein tumor thrombus. **(d)** The diffuse nature of ^90^Y bremsstrahlung SPECT/CT is unable to resolve the absence of activity within the portal vein tumor thrombus. **(e**, **f)** Four months post-radioembolization, a follow-up triphasic CT liver in the portal venous phase shows significant progression of portal vein tumor thrombosis, validating the ^90^Y PET/CT findings.

^90^Y PET/CT outperformed ^90^Y bremsstrahlung SPECT/CT in all aspects of qualitative analysis. Its image resolution was consistently superior to ^90^Y bremsstrahlung SPECT/CT (Figures 1 and 2), which also improved operator confidence in diagnostic reporting. Among 23 patients, 7 had tumor vascular thrombosis seen on diagnostic sectional imaging (Additional file 1: Table S4). ^90^Y PET/CT was able to provide conclusive information about the presence or absence of activity within targeted tumor vascular thrombosis in all seven patients (Figure 3), whereas ^90^Y bremsstrahlung SPECT/CT was conclusive in only three of seven patients; the remaining four patients were indeterminate due to its low resolution. In two patients without visually detectable activity on ^90^Y PET/CT within targeted tumor vascular thrombosis, follow-up scans confirmed disease progression in these lesions (Figure 4). Therefore, within the statistical limitations of our small dataset, ^90^Y PET/CT had a 100% accuracy for the provision of conclusive information about the presence or absence of activity within targeted tumor vascular thrombosis, compared to only 30% for ^90^Y bremsstrahlung SPECT/CT (60% sensitivity and positive predictive value; 0% specificity and negative predictive value).

**Figure 5:**
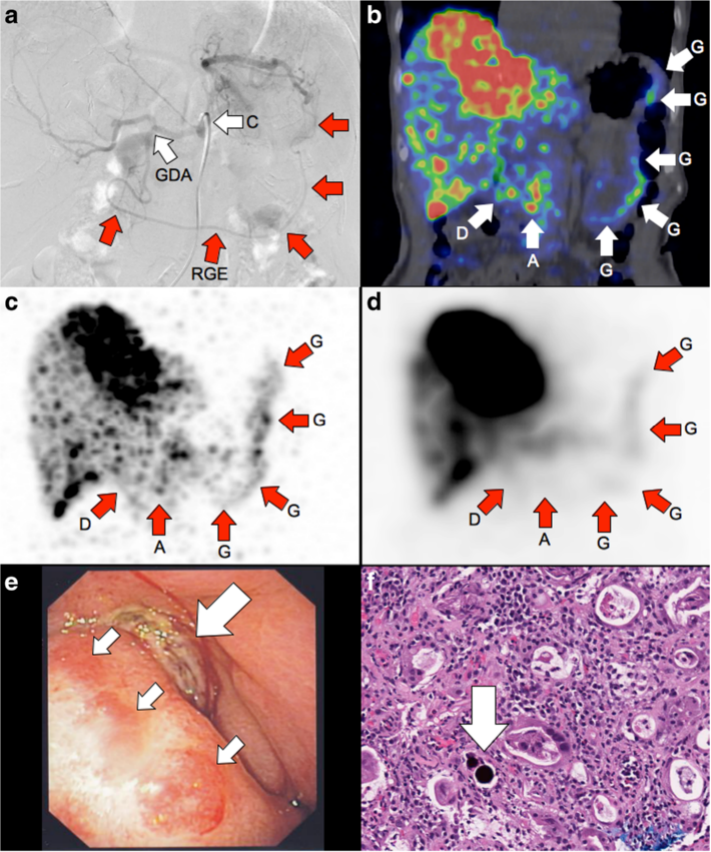
**Patient 17.**^90 ^Y radioembolization of a recurrent intra-hepatic cholangiocarcinoma. **(a)** Pre-radioembolization coeliac axis ‘C’ digital subtraction angiography (DSA) shows the common hepatic artery bifurcating into the right hepatic and gastroduodenal arteries ‘GDA’, later branching into the right gastroepiploic artery ‘RGE’. DSA at the conclusion of ^90^Y radioembolization demonstrated significant vascular stasis and reflux of contrast into the gastroduodenal and right gastroepiploic arteries (images not shown). **(b)** Coronal view of post-radioembolization ^90^Y PET/CT and its **(c)** MIP demonstrates in high resolution, non-target activity along the gastric greater curve ‘G’, antrum ‘A’ and proximal duodenum ‘D’. **(d)** MIP of ^90^Y bremsstrahlung SPECT shows concordant non-target activity, but of lower intensity and resolution. By quantification ^90^Y PET activity, detailed in Part 2 [38], the non-target mean absorbed doses to the gastric greater curve, gastric outlet, and proximal duodenum were approximately 49, 65, and 53 Gy, respectively. Within weeks, the patient developed persistent epigastric pain. **(e)** Gastroscopy at 3.3 months revealed extensive inflammation along the stomach and proximal duodenum. A Forrest III ulcer was present in the pylorus (large arrow). The ulcer edge (small arrows) was inflamed and indurated. **(f)** Photomicrograph (H&E stain; ×200) of the gastric biopsy showed edema, congestion, and diffuse lymphoplasmacytic, eosinophilic, and neutrophilic infiltrates. A purple-staining resin microsphere (arrow) surrounded by a halo was seen in the lamina propria. The surrounding gastric glands show flattening of the epithelium, dilatation, and reactive atypia, consistent with severe cellular injury.

**Figure 6:**
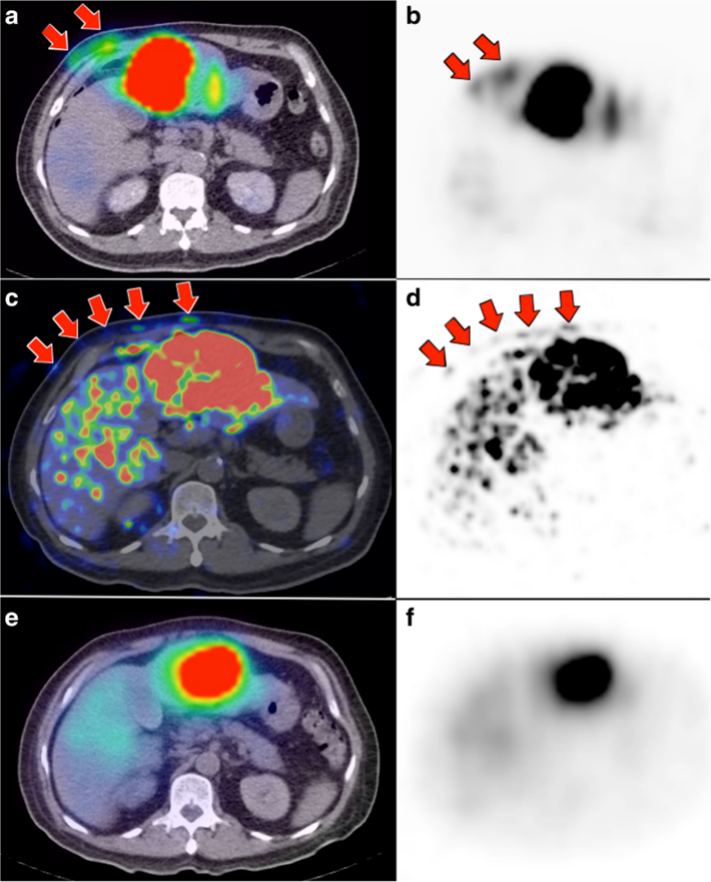
**Patient 22.**^90 ^Y radioembolization of part of a segment III HCC via the terminal branch of the right internal mammary artery (RIMA). **(a**, **b)** Pre-therapy ^99m^Tc MAA SPECT/CT demonstrates non-target activity in the right lower anterior chest wall (arrows), shunted from proximal branches of the RIMA. **(c**, **d)**^90^Y PET/CT depicts in high-resolution, non-target activity conforming to the anatomy of the right lower anterior chest wall, concordant with ^99m^Tc MAA SPECT/CT. The slight dissimilarity in chest wall activity biodistribution between ^90^Y PET/CT versus ^99m^Tc MAA SPECT/CT is due to local arterial flow changes after prophylactic bland embolization of proximal branches of the RIMA (images not shown). **(e**, **f)**^90^Y bremsstrahlung SPECT/CT was unable to detect any non-target activity in the chest wall due to its low image resolution.

**Figure 7:**
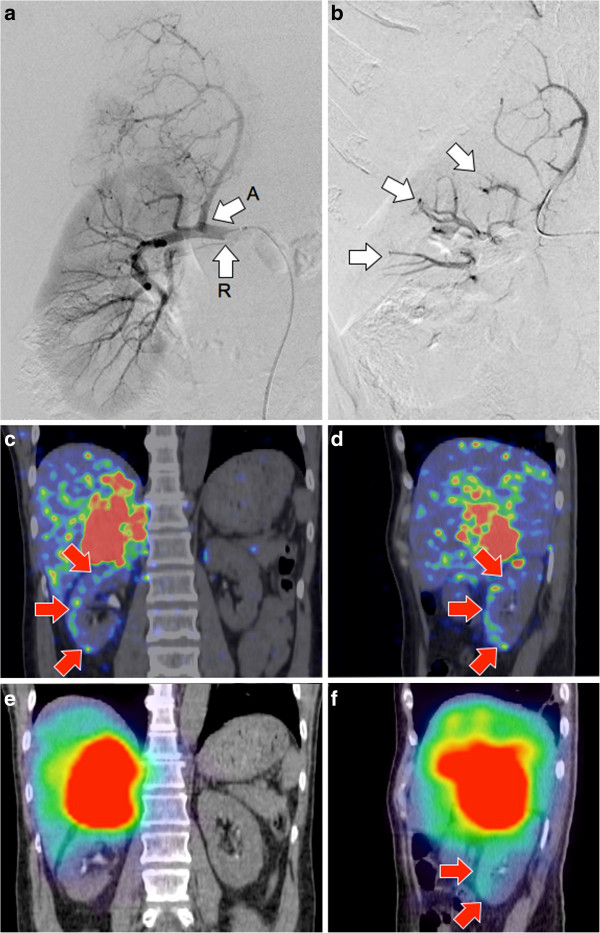
**Patient 9.**^90 ^Y radioembolization of an organ other than the liver. KIT-negative GIST with bulky metastasis to the right adrenal gland, refractory to tyrosine kinase inhibitors. **(a)** Pre-radioembolization digital subtraction angiogram (DSA) with catheter tip in the right renal artery ‘R’ demonstrates the origin of the right inferior adrenal artery ‘A’ and the arterial trees of the adrenal tumor and right kidney. The catheter tip was advanced deep into the right inferior adrenal artery for ^90^Y radioembolization. **(b)** Post-radioembolization DSA with no change in catheter tip position demonstrates significant vascular stasis and reflux of contrast down the right inferior adrenal artery and distally into the terminal branches of the right renal artery (arrows). **(c**, **d)**^90^Y PET/CT depicts in high resolution, non-target activity conforming to the anatomy of the right renal cortex (arrows). **(e**, **f)** On ^90^Y bremsstrahlung SPECT/CT, non-target activity was indistinguishable from adjacent activity bloom in the coronal plane, but seen as low-grade, diffuse activity in the saggital plane (arrows).

^90^Y PET/CT detected non-target activity in all eight of eight patients, whereas ^90^Y bremsstrahlung SPECT/CT detected only four of eight patients initially - a further three were seen only with hindsight of ^90^Y PET/CT findings (Additional file 1: Table S5). These eight cases include the stomach (Figure 5), duodenum, chest wall (Figure 6), kidney (Figure 7), untargeted liver, and gallbladder. All 23 patients underwent a retrospective review of clinical records to exclude any clinically significant non-target activity undetected by both imaging modalities. Three of 23 patients were non-residents who were discharged back to their home countries after ^90^Y radioembolization and were lost to follow-up. Of the remaining 20 patients, there were no cases of toxicity greater than or equal to CTCAE Grade 3 attributable to undetected non-target activity in extra-hepatic viscera at a median follow-up of 5.4 months (mean 6.3 months, 95% CI 4.5 to 8.1 months). Therefore, within the statistical limitations of our small dataset, ^90^Y PET/CT had a 100% accuracy for non-target activity detection, compared to 80% for ^90^Y bremsstrahlung SPECT/CT (50% sensitivity; 100% specificity and positive predictive value; 75% negative predictive value).

### Discussion

^90^Y is an imperfect PET tracer. However, its superior image resolution makes it very attractive for clinical use. With proper diagnostic reporting technique, ^90^Y PET/CT consistently out-performed ^90^Y bremsstrahlung SPECT/CT in high-resolution imaging to confirm technical success, detection of non-target activity, and providing conclusive information about ^90^Y activity within targeted tumor vascular thrombosis. These qualitative parameters have prognostic implications which may impact patient management early in the post-radioembolization period.

^90^Y radioembolization is complex, and continuity of care is paramount to the clinical utility of ^90^Y PET/CT. This is because case-specific issues occur throughout the planning-therapy continuum which can affect the final microsphere biodistribution, e.g., selective sparing of liver segments, vascular stasis, risk of microsphere reflux, vessels-at-risk, on-table changes to the intended vascular approach. The same attending nuclear medicine physician who follows through the entire planning-therapy continuum already has in mind an expected activity biodistribution when reviewing the ^90^Y PET/CT. This case-specific knowledge improves overall diagnostic accuracy and operator confidence, and leads to clinically meaningful reporting.

Despite the superior image resolution of ^90^Y PET/CT over ^90^Y bremsstrahlung SPECT/CT, parts of extra-hepatic viscera which lie closely adjacent to the liver still remain blind spots for non-target activity detection, e.g., gallbladder fundus, gastric lesser curve, pylorus, and proximal duodenum. These often appear anatomically inseparable from the adjacent liver on ^90^Y PET/CT, making assessment for non-target activity in these areas very challenging. This issue is further compounded by varying degrees of PET/CT mis-registration due to the relatively long ^90^Y PET acquisition time. However, these issues similarly affect ^90^Y bremsstrahlung SPECT/CT and should therefore not be viewed as a comparative disadvantage.

Despite background noise, ^90^Y PET/CT had a higher detection rate for non-target activity than ^90^Y bremsstrahlung SPECT/CT. Our counter-intuitive technique of deliberately increasing background noise levels has proven to be effective. Its rationale lies in our observation that noise spikes may be of higher visual intensity than non-target activity, as the latter depends on the quantity of shunted microspheres and its ^90^Y radioconcentration within the non-target arterial territory. This means that visually subtle but genuine trace non-target activity may appear less intense than background noise. Quantitative reconstruction methods proposed by Carlier et al. to distinguish signal from noise were not investigated in our study [32]. Therefore, from a qualitative perspective, a diagnosis of non-target activity should not be made on the basis of its visual intensity, but on its *pattern* and whether it *conforms* to underlying anatomy. The presence of a plausible vascular etiology will greatly lend support to the diagnosis, although this is not an essential criteria because it may not always be possible to identify a culprit vessel upon retrospective review of angiography.

The presence or absence of correlative clinical signs or symptoms do not feature in our criteria for non-target activity because clinical sequelae is a quantitative function of dose–response radiobiology over time, with no bearing on the qualitative presence of non-target activity at the time of scan. For example, it is common to detect subtle non-target activity in an untargeted liver lobe due to minor microsphere reflux or arterio-portal shunting, with no clinically relevant sequelae. Another example is the presence of mild non-target activity in the gallbladder wall, without ensuing clinical cholecystitis. It follows that the absence of correlative clinical signs or symptoms do not invalidate a ^90^Y PET/CT diagnosis of non-target activity.

The detection of non-target activity should immediately be followed by an assessment of the risk of developing clinically significant radiation toxicity. Accurate risk assessment facilitates appropriate action to be undertaken early to mitigate potential radiation injury, even if the patient is asymptomatic at the time of scan. This should be based on ^90^Y PET quantification of the non-target tissue absorbed dose, which is superior to subjective visual assessment alone. Exceptions are cases of visually subtle, trace non-target activity where the absorbed doses are unlikely to be clinically relevant. Similarly, it may sometimes be difficult to distinguish noise spikes from genuine ^90^Y activity. However, such indeterminate activity foci are usually too mild to result in any clinically relevant toxicity even if genuine, and therefore do not impact post-radioembolization management. The topic of ^90^Y PET quantification and its clinical applications in tissue dose–response assessment and tumor predictive dosimetry are presented in part 2 [38].

## Conclusions

Adherence to proper diagnostic reporting technique and emphasis on continuity of care are vital to the clinical utility of post-radioembolization ^90^Y PET/CT. With proper reporting technique, the presence of background noise did not pose a problem, and ^90^Y PET/CT consistently out-performed ^90^Y bremsstrahlung SPECT/CT in all aspects of qualitative analysis. ^90^Y PET/CT may supersede ^90^Y bremsstrahlung SPECT/CT as the modality-of-choice for post-radioembolization assessment of microsphere biodistribution.

## Competing interests

YHK, ASWG, KHT, and PKHC receive research funding from Sirtex Medical Singapore. ASWG and PKHC receive honoraria from Sirtex Medical Singapore.

## Authors’ contributions

YHK, YST, GKYL, SS, AEHT, DCEN, and ASWG were involved in study design, implementation, analysis, and manuscript preparation. JDS, JY, and DWT were involved in study design, scan optimization, and manuscript preparation. AT was involved in histopathological analysis and manuscript preparation. PKHC was involved in clinical care and manuscript preparation. MCB, FGI, TKBT, TNY, AG, RHGL, KHT and BST were involved in radioembolization, angiographic analysis, and manuscript preparation. All authors read and approved the final manuscript.

## Supplementary Material

Additional file 1 Online resource. Click here for file
