# Supplementary material for: Post-radioembolization yttrium-90 PET/CT - part 1: diagnostic reporting
Source: EJNMMI Res. 2013 Jul 25;3:56. doi: 10.1186/2191-219X-3-56 (PMC3726297; doi:10.1186/2191-219X-3-56)
Supplement: Additional file 1 — Online resource. [file 2191-219X-3-56-S1.doc]

**ONLINE RESOURCE**

**TITLE**

Post-radioembolization yttrium-90 PET/CT. Part 1: Diagnostic reporting

**FIRST AUTHOR CONTACT**

Dr Yung Hsiang KAO

Department of Nuclear Medicine, Austin Hospital

Level 1, Harold Stokes Building, 145 Studley Road, Heidelberg

Melbourne, Victoria 3084, AUSTRALIA

Telephone: +61 3 94965718; Fax: +61 3 94576605

Email: yung.h.kao@gmail.com

**TABLE OF CONTENTS**

1. ^90^Y PET/CT protocol (page 2)
2. Online Resource Table 1 - Patient and disease characteristics (page 3)
3. Online Resource Table 2 - ^90^Y radioembolization characteristics (page 4)
4. Online Resource Table 3 - Optimal PET visual display thresholds for target and non-target ^90^Y activity detection (page 5)
5. Online Resource Table 4 - ^90^Y bremsstrahlung SPECT/CT versus ^90^Y PET/CT in providing conclusive information about ^90^Y activity in targeted tumor vascular thrombosis (page 6)
6. Online Resource Table 5 - ^90^Y bremsstrahlung SPECT/CT versus ^90^Y PET/CT to detect non-target ^90^Y activity (page 7)

**^90^Y PET/CT PROTOCOL**

Scanner: GE Discovery™ 690 PET/CT, General Electric Medical Systems, Milwaukee, WI, USA.

PET detector: 24 rings of 576 cerium-activated lutetium-yttrium-orthosilicate (LYSO) crystals; ring diameter 81cm.

PET acquisition: ^90^Y positron fraction setting 3.186x10^-5^; ^90^Y half-life setting 64.1 hours; 15 minutes per bed position; 2 to 3 bed positions from the diaphragm downwards to cover the entire liver; 3cm overlap between adjacent bed positions.

PET image reconstruction: *‘VUE Point FX with Sharp-IR’* time-of-flight 3D-OSEM iterative reconstruction; 3 iterations 18 subsets; filter cut-off 6.0 mm; Z-axis filter ‘*Heavy*’; matrix 192x192; axial field-of-view 15.7cm; trans-axial field-of-view 70cm.

CT: 64-slice; 140kV; 70-400mA; 3.75mm slice thickness; slice interval 3.27mm; full helical tube rotation 0.8 seconds; matrix 192x192.

**ONLINE RESOURCE TABLE 1**

**Optimal PET visual display thresholds for target and non-target ^90^Y activity detection**

| **Patient No.** | **Target activity detection:**  **Optimal PET visual display threshold, kBq/ml (%)** | **Location of**  **non-target activity** | **Non-target activity detection:**  **Optimal PET visual display threshold, kBq/ml (%)** |
| --- | --- | --- | --- |
| 1 | 7,000 (51.0) | N/A | N/A |
| 2 | 7,000 (56.0) | N/A | N/A |
| 3 | 6,000 (56.5) | N/A | N/A |
| 4 | 7,000 (64.0) | N/A | N/A |
| 5 | 5,000 (74.0) | N/A | N/A |
| 6 | 9,000 (34.0) | N/A | N/A |
| 7 | 7,000 (52.5) | Untargeted liver * | 500 (4.0) |
| 8 | 5,000 (32.5) | N/A | N/A |
| 9 | 8,000 (41.5) | Right kidney † | 2,000 (10.0) |
| 10 | 9,000 (29.5) | Untargeted liver * | 2,000 (7.0) |
| 11 | 6,000 (57.5) | N/A | N/A |
| 12 | 6,000 (47.0) | N/A | N/A |
| 13 | 6,000 (58.5) | N/A | N/A |
| 14 | 8,000 (33.0) | Gastric wall † | 1,000 (4.0) |
| 15 | 7,000 (67.5) | N/A | N/A |
| 16 | 7,000 (55.0) | Gallbladder † | 3,000 (24.0) |
| 17 | 6,000 (97.0) | Gastric and duodenal wall * | 4,000 (65.0) |
| 18 | 3,000 (39.0) | N/A | N/A |
| 19 | 5,000 (47.5) | N/A | N/A |
| 20 | 6,000 (91.5) | Untargeted liver * | 3,000 (46.0) |
| 21 | 16,000 (52.0) | N/A | N/A |
| 22 | 5,000 (45.0) | Chest wall ‡ | 2,000 (18.0) |
| 23 | 8,000 (63.0) | N/A | N/A |

Untargeted liver: ^90^Y activity in untargeted liver may be due to microsphere reflux, arterio-portal shunting, or both; ***** Detected by both ^90^Y bremsstrahlung SPECT/CT and ^90^Y PET/CT ; † Detected by both ^90^Y bremsstrahlung SPECT/CT and ^90^Y PET/CT, but non-target activity on ^90^Y bremsstrahlung SPECT/CT was only seen in hindsight after its detection on ^90^Y PET/CT; ‡ Undetectable by ^90^Y bremsstrahlung SPECT/CT

**ONLINE RESOURCE TABLE 2**

**Patient and disease characteristics**

| **Patient No.** | **Sex** | **Age** | **Disease** | **Tumor vascular thrombosis** | **Lung shunt**  **fraction (%)** |
| --- | --- | --- | --- | --- | --- |
| 1 | F | 67 | HCC | No | 11.3 |
| 2 | M | 60 | HCC | No | 2.0 |
| 3 | M | 64 | HCC | No | 2.6 |
| 4 | M | 62 | HCC | Portal vein | 8.2 |
| 5 | F | 40 | Pancreatic neuroendocrine | No | 7.7 |
| 6 | M | 77 | HCC | Inferior vena cava | 7.9 |
| 7 | M | 63 | HCC | No | 24.2 |
| 8 | M | 77 | HCC | No | 3.6 |
| 9 | M | 51 | Adrenal metastatic GIST | No | 6.3 |
| 10 | M | 63 | HCC | Portal vein | 4.1 |
| 11 | M | 68 | HCC | No | 2.1 |
| 12 | M | 75 | HCC | Portal vein | 7.1 |
| 13 | F | 65 | HCC | Portal vein | 9.0 |
| 14 | M | 65 | HCC | Portal vein | 4.2 |
| 15 | M | 57 | HCC | Portal vein | 5.9 |
| 16 | F | 68 | HCC | No | 4.6 |
| 17 | F | 67 | Cholangiocarcinoma | No | 6.5 |
| 18 | M | 52 | HCC | No | 14.3 |
| 19 | M | 62 | HCC | No | 1.5 |
| 20 | M | 59 | Cholangiocarcinoma | No | 4.8 |
| 21 | F | 68 | HCC | No | 2.6 |
| 22 | M | 70 | HCC | No | 4.8 |
| 23 | F | 59 | HCC | No | 5.8 |

HCC: hepatocellular carcinoma; GIST: gastrointestinal stromal tumor

**ONLINE RESOURCE TABLE 3**

**^90^Y radioembolization characteristics**

| **Patient**  **No.** | **No. of arterial territories** | **Arterial territories** | **Injected ^90^Y activity**  **GBq (mCi) *** | **Interval between radioembolization to ^90^Y PET/CT (hr:min)** |
| --- | --- | --- | --- | --- |
| 1 | 2 | Dominant branch and  segment IVB branch of RHA | 4.08 (110.3) | 21:43 |
| 2 | 1 | Posterior branch of RHA | 2.66 (71.8) | 21:20 |
| 3 | 1 | RHA | 3.08 (83.2) | 17:04 |
| 4 | 1 | Replaced RHA | 2.29 (62.0) | 19:52 |
| 5 | 2 | PHA; additional boost to RHA | 1.96 (52.9) | 23:10 |
| 6 | 2 | RIPA; RHA | 4.64 (125.3) | 18:36 |
| 7 | 1 | LHA | 1.67 (45.0) | 22:08 |
| 8 | 1 | PHA | 1.13 (30.6) | 20:55 |
| 9 † | 2 | Right inferior adrenal artery;  replaced RHA | 2.86 (77.2) | 4:44 |
| 10 | 1 | Anterior branch of RHA | 3.06 (82.8) | 22:07 |
| 11 | 2 | LHA; RHA | 1.43 (38.6) | 21:50 |
| 12 | 2 | RHA; branch to segment IV | 2.91 (78.6) | 23:52 |
| 13 | 1 | RHA | 1.49 (40.2) | 23:10 |
| 14 | 2 | LHA; RHA | 1.44 (38.9) | 5:33 |
| 15 | 3 | LHA; MHA; RHA | 2.02 (54.6) | 17:54 |
| 16 | 1 | RHA | 2.47 (66.8) | 20:44 |
| 17 ‡ | 1 | RHA | 2.11 (57.0) | 21:08 |
| 18 | 2 | RIPA; PHA | 2.68 (72.3) | 21:21 |
| 19 | 3 | RIPA; LHA; RHA | 5.02 (135.6) | 23:47 |
| 20 | 1 | RHA | 1.15 (31.2) | 24:02 |
| 21 | 1 | RHA | 1.05 (28.3) | 6:03 |
| 22 | 4 | Right internal mammary artery; LHA; MHA; RHA | 2.18 (59.0) | 18:22 |
| 23 | 2 | LHA; RHA | 1.45 (39.2) | 22:23 |

RHA: right hepatic artery; RIPA: right inferior phrenic artery; LHA: left hepatic artery; PHA: proper hepatic artery; MHA: middle hepatic artery; * Measured by dose calibrator; † Chemo-refractory bulky right adrenal GIST metastasis with additional liver segment IV metastasis; ‡ Technically unsuccessful due to significant non-target gastric wall microsphere shunting causing CTCAE Grade 3 toxicity

**ONLINE RESOURCE TABLE 4**

**^90^Y bremsstrahlung SPECT/CT vs ^90^Y PET/CT in providing conclusive**

**information about ^90^Y activity in targeted tumor vascular thrombosis**

| **Patient**  **No.** | **Reference for presence or absence of ^90^Y activity in targeted tumor vascular thrombosis** | **Was ^90^Y bremsstrahlung SPECT/CT able to provide conclusive information?** | **Was ^90^Y PET/CT able to provide conclusive information?** |
| --- | --- | --- | --- |
| 4 | Present * | Yes | Yes |
| 6 | Present † | No | Yes |
| 10 | Present * | Yes | Yes |
| 12 | Absent ‡ | No | Yes |
| 13 | Present † | No | Yes |
| 14 | Present * | Yes | Yes |
| 15 | Absent ‡ | No | Yes |

* Concordant findings by both ^90^Y bremsstrahlung SPECT/CT and ^90^Y PET/CT; † ^90^Y bremsstrahlung SPECT/CT was indeterminate due to low image resolution, but conclusively present on ^90^Y PET/CT;

‡ ^90^Y bremsstrahlung SPECT/CT was indeterminate due to low image resolution, but conclusively absent on ^90^Y PET/CT and clinically validated by follow-up diagnostic CT confirming the progression of targeted tumor vascular thrombosis

**ONLINE RESOURCE TABLE 5**

**^90^Y bremsstrahlung SPECT/CT vs ^90^Y PET/CT to detect non-target ^90^Y activity**

| **Patient No.** | **Location of non-target ^90^Y activity** | **Non-target ^90^Y activity detected**  **on ^90^Y bremsstrahlung SPECT/CT** | **Non-target ^90^Y activity detected on ^90^Y PET/CT** |
| --- | --- | --- | --- |
| 7 | Untargeted liver | Yes | Yes |
| 9 | Right kidney | No * | Yes |
| 10 | Untargeted liver | Yes | Yes |
| 14 | Gastric wall | No * | Yes |
| 16 | Gallbladder | No * | Yes |
| 17 | Gastric & duodenal wall | Yes | Yes |
| 20 | Untargeted liver | Yes | Yes |
| 22 | Chest wall | No | Yes |

* Non-target activity seen on ^90^Y bremsstrahlung SPECT/CT only with hindsight of ^90^Y PET/CT findings
